# Cancer patients’ experiences with and opinions on the process 'Screening of Distress and Referral Need' (SDRN) in clinical practice: A quantitative observational clinical study

**DOI:** 10.1371/journal.pone.0198722

**Published:** 2018-06-14

**Authors:** Floor M. van Nuenen, Stacey M. Donofrio, Harry B. M. van de Wiel, Josette E. H. M. Hoekstra-Weebers

**Affiliations:** 1 Wenckebach Institute, University Medical Center Groningen, University of Groningen, Groningen, the Netherlands; 2 Department of Psychology, University of Groningen, Department of Psychology, Groningen, the Netherlands; 3 Netherlands Comprehensive Cancer Organization, Groningen, the Netherlands; University of Zurich, SWITZERLAND

## Abstract

**Objective:**

This observational clinical study investigated patients’ experiences with and opinions on the Dutch ‘Screening of Distress and Referral Need’ (SDRN) process implemented in oncology practice. Insight into these can guide improvement of the SDRN process.

**Methods:**

Patients from hospitals that had implemented SDRN for at least a year completed questions on experiences with essential SDRN process steps (1: completion of the Distress Thermometer and Problem List as screening instrument (DT&PL), 2: information on SDRN+DT&PL, 3: information on referral options, 4: discussing DT&PL responses, 5: referral when needed), and on opinions about SDRN and DT&PL. Descriptive and univariate analyses were conducted.

**Results:**

Of the 498 participants (response = 54%), 81% completed a DT&PL, of whom 86–87% was exposed to steps 2–3 and 76% discussed responses; only three needing care were not offered referral. Sixty-one percent encountered all SDRN steps and 78% would recommend SDRN to others. Recommending SDRN is related to more frequent DT&PL completion (t = -2.5; p≤0.01), receipt of information on SDRN+DT&PL and referral options (X^2^ = 4.9; p≤0.05 and X^2^ = 5.9; p≤0.05 respectively), discussion of responses (X^2^ = 10.2; p≤0.001), and fuller exposure to SDRN process steps (X^2^ = 14.8; p≤0.01). Percentages (strongly) agreeing were highest on the DT&PL being useful (90%) and suitable (88%), and lowest on burdensome (31%) and time-consuming (28%).

**Conclusion:**

The majority of participating patients encountered the steps of the SDRN process considered essential, with 3/5 having encountered all steps. Referral is largely targeted to patients’ need. Patients’ perceived benefit of SDRN increases with fuller exposure to all process steps. Therefore, improvements, particularly in DT&PL completion and discussion of responses should be made.

## Introduction

Distress screening was developed to decrease the discrepancy between the percentage of cancer patients experiencing clinically-elevated distress for which professional psychosocial care is warranted (percentages reported range between 25–50% [1,2]) and the percentage of patients actually receiving such care (percentages reported vary between 7–24% [3,4]). Regular screening of level and nature of distress during cancer treatment and follow-up may facilitate appropriate and timely referral to psychosocial and/or allied healthcare professionals, and thus ensure that patients needing additional care receive such care before distress becomes overwhelming.

Randomized controlled trials (RCTs) mostly focus on the effect of a distress screening process on patient-reported outcomes such as quality of life, patient-doctor communication, or on outcomes such as referrals or medical consumption. Results reported are inconsistent and vary from a positive to no effect [5–13]. Studies examining a more extensive process including triage more often found positive results [5,6,8,9], in contrast to studies investigating a process consisting of giving completed patient-reported outcome measures to a healthcare provider who decided independently what to do with the results [7,10–13]. This suggests that triage should be part of a distress screening procedure. It remains unclear from the studies using triage whether communication about responses on a screening instrument took place at all, only with patients who scored above a cutoff, or with all patients.

The literature on patients’ opinions on a distress screening procedure or on the instrument used is sparse, although patients are supposed to benefit from distress screening. According to three (randomized) controlled trials, between 87–99% of patients thought that a screening procedure would have added value in oncologic practice [14–16]. Four studies conducted in a routine clinical setting reported that 49–98% of patients replied that the instrument used for screening was useful [17–20]. Only one study examined patients’ opinions on the Distress Thermometer (DT) in combination with the Problem List (PL) [20], although this instrument is often used for screening worldwide.

In the Netherlands, a process ‘Screening for Distress and Referral Need’ (SDRN) was developed including the following steps: 1) regular *completion* of the Dutch DT&PL [21] during curative or palliative treatment and follow-up, 2) *discussion* of the responses between care provider and patient, regardless of whether the DT-score is below or above the cut-off, and 3) *referral* if needed or wished by the patient to a psychosocial and/or allied healthcare provider depending on the nature of the problems experienced [22,23]. Additionally, we considered providing 4) information to patients about the goal of SDRN and the DT&PL and 5) about referral options and the specific expertise of psychosocial and allied healthcare professionals to be essential. SDRN has been implemented in routine clinical practice in the majority of the hospitals in the north and east of the Netherlands for at least one group of patients, mostly breast cancer patients. Health care providers of these hospitals find the implementation of this process feasible [24]. However, the extent to which patients experience the different steps in this process in daily oncology practice is unknown and information on patients’ opinions on a screening process is very limited. It may well be that patients who are exposed to relevant steps in an SDRN process have a more positive opinion of the instrument used and perceive the process as more relevant to the quality of the care they receive.

Therefore, this observational clinical study examines: 1) patients’ experiences with the above-mentioned essential steps of the Dutch SDRN process in clinical practice; 2) patients’ opinions on SDRN and on the DT&PL as screening instrument; 3) and relationships between patients’ experiences and opinions.

## Materials and methods

### Patients

Oncology patients visiting a surgical, medical, gynaecological or urological outpatient department from ten Dutch hospitals that had implemented the SDRN process in routine clinical practice for at least one year, who were aware of their treatment plan, cognitively and physically able to complete a questionnaire, ≥18 of age, and sufficiently fluent in Dutch were eligible for study participation. Hospitals were located in the northeastern part of the Netherlands, the catchment area of the Netherlands Comprehensive Cancer Organization, location Groningen (IKNL-G). IKNL-G supported SDRN implementation in these hospitals and provided centralized project management [24]. Patients from the first 10 of the 21 hospitals in the IKNL-G area that completed SDRN implementation for at least one group of patients for at least a year were invited to participate in the study. Three hospitals had implemented SDRN for breast cancer patients only. One hospital had implemented SDRN for prostate and breast cancer patients. The remaining hospitals had implemented SDRN for patients with breast, digestive, prostate and/or other types of cancer.

### Procedure

The medical specialist or nurse informed all eligible patients visiting an outpatient clinic about the study and invited them to participate. Eligible patients were informed that SDRN had been implemented and that we invited them in a study assessing their experiences with SDRN in the hospital. Patients received a package including written information about the study, the questionnaire, an informed consent form and a pre-franked return envelope in which they could return the completed questionnaire to the IKNL-G. In eight hospitals 100 packages were handed out, in one hospital 50 and in the last 66, because the number of new cancer patients in the last two hospitals was lower (Dutch Cancer Registry). Handing out of packages took 2–4 weeks. IKNL-G coordinated the study. No information was given by the hospitals about the patients approached for participation for reasons of anonymity. Consequently, non-responders were not mailed or phoned to remind them to send a completed questionnaire to IKNL-G. The medical ethical committee of the University Medical Centre Groningen exempted the study from full review; no formal approval was needed according to the Dutch Medical Research Involving Human Subjects Act. The study was performed according to the Helsinki Declaration and the ethics committees of the participating hospitals.

### Instrument

Patients were asked whether they completed a DT&PL since their diagnosis. Those who answered ‘yes’ responded to a further seven self-developed questions assessing their experiences with SDRN (questions 1–5), and their opinions on the DT&PL (question 6) and SDRN (question 7). Patients were asked to indicate: 1) how often they completed a DT&PL; 2) if they received information about the purpose of SDRN and the DT&PL; 3) if they received information about referral options and the expertise of different healthcare providers; 4) if they discussed responses with a care provider, and if yes, with whom and their satisfaction with the discussion; 5) if they were offered referral according to their need; 6) their agreement on eleven statements about the DT&PL; and 7) if they would recommend SDRN to other cancer patients as a proxy of their general opinion of SDRN as a process (questions and response options in S1 File).

Additionally, patients completed questions on the following socio-demographic and illness-related characteristics; age, gender, education level (low (elementary or low vocational school), middle (secondary or middle vocational education), high (high vocational or university)), cancer type, date of diagnosis, treatment modalities, and treatment phase (watchful waiting, receiving active treatment, follow-up). A medical specialist placed patients in a curative and palliative treatment group based on their cancer type and treatment.

### Analyses

Descriptive analyses were computed to examine patients’ answers to the questions. Independent t-tests (continuous variables) and chi-square tests (categorical variables) were performed to investigate differences between respondents who completed a DT&PL since their cancer diagnosis and those who did not. We conducted independent samples t-tests, Mann-Whitney U-tests and Spearman’s rho correlations (continuous variables), and chi-square and Fisher exact tests (categorical variables) to examine univariate relationships between variables. Treatment modalities were dichotomized into non-intensive treatment (watchful waiting, surgery only, and radiotherapy only) and intensive treatment (the remaining modalities) for analysis because of the low numbers in some of the categories. All tests were two-sided. We used SPSS, version 22, for analyses.

## Results

Of the 916 questionnaires distributed, 498 were returned (response = 54%). Response was comparable between hospitals. Table 1 shows information on respondents’ socio-demographic and illness-related characteristics. Of the respondents, 78% were women and 24% had completed low level education. Mean age was 59.8 years. Sixty-seven percent had breast cancer and mean time since diagnosis was 1.2 years. Respondents received different types of treatment, of whom 78% intensive treatment, 92% received treatment with a curative intent, and 46% was under active treatment.

**Table 1:** Socio-demographic and illness-related characteristics and comparison between respondents who completed a DT&PL after diagnosis and those who did not.

Characteristics	Total group (n = 498)	Completed a DT&PL (n = 398)^§^	Did not complete a DT&PL (n = 94)^§^	Comparison between groups
Age				
Mean ± SD	59.8±10.8	59.4±10.8	61.2±10.237.	t = 1.4^ns^
Range	29.9–87.5	29.9–87.5	1–85.1	
Gender (N(%))				
Men	111(22)	77(19)	32(34)	X^2^ = 9.5**
Women	387(78)	321(81)	62(66)	
Education (N(%))				
Low	116(24)	96(25)	17(19)	X^2^ = 1.6^ns^
Middle	232(48)	185(47)	46(50)	
High	139(29)	109(28)	29(32)	
Type of cancer (N(%))				
Breast	333(67)	272(68)	57(61)	X^2^ = 15.5**
Prostate	48(10)	28(7)	19(20)	
Digestive	32(6)	27(7)	5(5)	
Other	85(17)	71(18)†	13(14)††	
Time since diagnosis in years				
Mean ± SD	1.2±1.9	1.1±1.7	1.7±2.5	t = 2.5*
Range	0.0–19.4	0.0–18.9	0.0–19.4	
Treatment modality (N(%))				
Watchful waiting	9(2)	8(2)	1(1)	X^2^ = 11.8^ns^
Surgery	80(16)	65(17)	14(15)	
Surgery + radiotherapy	98(20)	71(18)	25(27)	
Surgery + chemotherapy	82(17)	65(17)	15(16)	
Surgery + radiotherapy + chemotherapy	138(28)	119(30)	19(20)	
Surgery + immunotherapy and/or hormonal therapy	20(4)	17(4)	3(3)	
Radiotherapy	18(4)	10(3)	7(8)	
Chemotherapy	30(6)	24(6)	6(7)	
Radiotherapy + chemotherapy	19(4)	16(4)	3(3)	
Treatment intensity (N(%))				
Non-intensive	107(22)	83(21)	22(24)	X^2^ = 0.3^ns^
Intensive	387(78)	312(79)	71(76)	
Treatment intent (N(%))				
Curative	452(92)	360(91)	86(93)	X^2^ = 0.2^ns^
Palliative	42(9)	35(9)	7(8)	
Treatment phase (N(%))				
Watchful waiting	9(2)	8(2)	1(1)	X^2^ = 2.8^ns^
Under active treatment	227(46)	188(48)	37(40)	
Follow-up	253(52)	194(50)	55(59)	

^§^ N varied somewhat due to missing data

† = 22 hematologic, 18 lung, 14 gynaecologic, 6 sarcoma/bone, 5 skin, 3 liver, 1 urologic, 2 unspecified;

†† = 2 hematologic, 1 lung, 1 gynaecologic, 2 sarcoma/bone, 4 skin, 2 head/neck, 1 brain;

ns = not significant;

*p≤0.05;

**p≤0.01

No information was provided by the physician or nurse about patients approached for study participation to the researchers. Consequently, participants could not be compared to non-participants.

Regarding the first SDRN step, 81% (N = 398) of the patients answered that they had completed a DT&PL in clinical practice and 19% (N = 94) (6 missing) that they had not done so. Comparison of those who did and those who did not complete a DT&PL showed significant differences in gender, cancer diagnosis and time since diagnosis (Table 1). The percentages of men and of prostate cancer patients were lower and time since diagnosis was shorter in the group that completed a DT&PL. Twenty-eight of the 77 (36%) men who completed a DT&PL had prostate cancer versus 19 of the 32 (59%) men who did not complete a DT&PL. Excluding the prostate cancer patients from analysis, no gender difference was found between those who completed a DT&PL and those who did not (X^2^ = 0.9, p = 0.4).

The following results relate to the 398 respondents who completed a DT&PL since diagnosis.

### Patients’ experiences with SDRN (Table 2)

Of these 398 respondents, 36% completed the DT&PL once, 35% twice, 20% three times, and 9% between four and eight times. Eighty-six percent received information on the purpose of SDRN and the DT&PL, and 87% received information about referral options and the expertise of psychosocial and allied healthcare professionals.

**Table 2:** Descriptives on study questions 1–5 and on variable created post-hoc, and univariate effects on respondents’ recommendation (question 7).

		7) Recommend others? N(%)		Univariate test
Questions 1–5 and variable created post-hoc	Total group N(%)	Yes 280(78)	No 80(22)	
1) How often did you complete the DT&PL?				
Once	132(36)			
Twice	130(35)			
Three times	74(20)			
Between four and eight times	34(9)			
Missing	28			
Mean±SD	2.1±1.1	2.2±1.1	1.8±0.8	t = -2.5**
2) Received information on (purpose of) SDRN and DT&PL?				
Yes	332(86)	245(89)	64(80)	X^2^ = 4.9*
No	53(14)		16(20)	
Missing	13	29(11)		
3) Received information about referral options and expertise?				
Yes	334(87)	247(89)	63(79)	X^2^ = 5.9*
No	50(13)		17(21)	
Missing	14	30(11)		
4) Response pattern discussed?				
Yes	289(76)	223(80)	50(64)	X^2^ = 10.2***
No	94(24)		29(36)	
Missing	15	54(20)		
4a) If yes, with whom				
Nurse	272(95)			
Medical specialist	6(2)			
Another healthcare provider	7(3)			
Missing	4			
4b) If yes, satisfaction with discussion				
Very satisfied (1)	89(32)			
Satisfied (2)	178(63)			
Moderately satisfied (3)	13(5)			
Dissatisfied (4)	1(0.4)			
Missing	8			
Mean±SD	1.7±0.6	1.7±0.5	2.0±0.7	t = 3.4***
5) Referral according to need?				
Offered but not needed	164(57)	125(56)	28(54)	Fisher’s exact = 5.0^ns^
Not offered and not needed	71(25)		15(29)	
Offered and needed	51(18)	53(24)	7(14)	
Not offered but needed	3(1)	43(19)	2(4)	
Missing	109	1(1)		
**Extent of exposure to SDRN**				
1) DT&PL only	18(5)	6(2)	4(5)	X^2^ = 14.8**
2) DT&PL+info SDRN/DT&PL or info referral options/expertise	40(10)	23(8)	10(13)	
3) DT&PL+info SDRN/DT&PL and info referral options/expertise	51(13)	28(10)	16(20)	
4) DT&PL+info SDRN/DT&PL or info referral+discussion	48(12)	30(11)	13(16)	
5) DT&PL+info SDRN/DT&PL+info referral+discussion	241(61)	193(69)	37(46)	

ns = not significant;

*p≤0.05;

**p≤0.01;

***p≤0.001

info = information

% do not always add up to 100 due to rounding

DT&PL responses were discussed with 76% of the respondents. Of these, 93% indicated that the nurse discussed responses with them, and 95% was (very) satisfied with this discussion.

Fifty-four respondents (19%) indicated that they needed care, of whom only three patients (6% (1% of total group)) were not offered a referral. Of the 289 respondents who completed the referral question, eight (3%) answered that the DT&PL was not discussed with them (2 missing), while of the 109 respondents who did not answer it, 90% indicated that responses were not discussed (X^2^ = 292.6; p≤0.001).

### Patients opinions on SDRN and the DT&PL

Of the respondents who completed a DT&PL in the clinic, 78% would recommend SDRN to other cancer patients, while 22% would not (38 missing) (Table 2).

Regarding the first eight statements on the DT&PL, the strongest agreement was found with the DT&PL being: ‘suitable for its purpose’ and ‘useful for my care provider’. The strongest disagreement was found with the statement that DT&PL completion is ‘time-consuming’. Concerning the three statements on patients’ perceptions on the purpose of DT&PL completion, the strongest agreement was found with the statement that it ‘offers insight into the problems I experience’ (Table 3).

**Table 3:** Descriptives on the DT&PL opinion statements (question 6), and univariate effects of opinions on information received, responses discussed, satisfaction with discussion, and recommending others.

6) DT&PL completion:	1 Agree strongly (N(%))	2 Agree (N(%))	3 Disagree (N(%))	4 Disagree strongly (N(%))	Missing (N)	Received information on SDRN and DT&PL? (z-value)	Responses discussed? (z-value)	Satisfaction with communication (Spearman’s ϱ)	Recommend others? (z-value)
Is pleasant	88(30)	135(46)	47(16)	26(9)	102	3.4***	3.3***	0.28***	6.8***
Is easy	132(44)	106(35)	44(15)	20(7)	96	1.2	1.3	0.14*	4.7***
Is burdensome	15(5)	74(26)	64(22)	136(47)	109	-1.9	-1.0	-0.23***	-3.8***
Is useful for myself	99(33)	107(36)	54(18)	38(13)	100	0.6	3.7***	0.19**	7.4***
Is difficult	28(10)	58(20)	67(23)	142(48)	103	-2.6**	-0.4	-0.09	-1.5
Is time-consuming	24(8)	58(20)	60(21)	147(51)	109	-3.9***	-2.1*	-0.1	-4.3***
Is suitable for its purpose	184(59)	91(29)	22(7)	15(5)	86	0.7	0.7	0.21***	6.8***
Is useful for my care provider	175(56)	108(34)	13(4)	19(6)	83	1.4	1.7	0.23***	6.2***
Offers me insight into problems I experience	85(24)	149(43)	56(16)	58(17)	50	2.5*	2.6**	0.24***	7.7***
Helps in communication with care provider	83(25)	115(35)	63(19)	70(21)	67	2.2*	4.5***	0.24***	7.4***
Gives me insight into problem severity	72(21)	115(34)	69(21)	79(23)	63	1.4	3.1**	0.19**	6.6***

*p≤0.05;

**p≤0.01;

***≤0.001;

% does not always add up to 100 due to rounding off

### Relationships between patients’ experiences and their opinions

#### Regarding SDRN

Those who would recommend SDRN had completed a DT&PL significantly more often than those who would not recommend SDRN. A significantly higher percentage of those who would recommend SDRN had received information about the purpose of SDRN and the DT&PL, and about referral options and the expertise of different healthcare disciplines, had discussed responses with a care provider, and they were significantly more satisfied with the discussion of the responses than those who would not recommend SDRN. Referral according to need did not significantly affect SDRN recommendation (Table 2). Because of the low numbers in two cells an additional comparison was performed between those needing care and those not needing care. No differences were found between these two groups.

A significantly higher percentage of patients who would recommend SDRN to others was exposed to the full SDRN process (Table 2).

#### Regarding DT&PL

Mann-Whitney tests showed five differences between respondents who received information about SDRN and the DT&PL and those who did not, namely the first group perceived completing the DT&PL as significantly more ‘pleasant’, and less ‘difficult’ and ‘time-consuming’ than patients who did not receive information. Additionally, the first group agreed significantly more that completing the DT&PL ‘offers insight into the problems they experience’ and that it ‘helps in the communication with their care provider’. Significant differences were found on six of the 11 DT&PL statements between patients who discussed responses with their care provider and those who did not. Satisfaction with the discussion correlated significantly, but weakly, with nine DT&PL statements. Respondents who would recommend SDRN to others were significantly more positive on ten of the 11 DT&PL statements (Table 3).

## Discussion

The present quantitative observational clinical study explored patients’ experiences with the different essential steps of the Dutch Screening for Distress and Referral Need process implemented long-term in everyday oncology practice. Additionally, their opinions on this process and the DT&PL were examined. Overall, a majority of participating patients encountered one or more of the steps considered essential in the Dutch SDRN process and, with the exception of three patients, referral was according to need. Patients exposed to essential SDRN procedure steps are more positive on SDRN and the DT&PL. Fuller exposure is related with higher positivity; patients who experienced more of the SDRN process are more likely to recommend it to others.

Regarding patients’ experience with the first step in the SDRN process, we found that more than four-fifths of the respondents had completed a DT&PL since they were diagnosed with cancer. Given that SDRN had been implemented for at least a year in participating hospitals, the percentage of patients who completed a DT&PL was lower than we expected. Compared to three recent studies, reporting percentages varying between 40–73% [25–27], our finding is high, but it is lower than that reported in a fourth study (94%) [28]. Similarly to our study, three of these studies examined the situation in oncology practice after a systematic implementation process [26–28]. We found that some patients did not complete the DT&PL. It may be that the instrument was not well introduced for certain patient groups; patients may not have been asked to complete a DT&PL, possibly because of shortage of time [24]. We found that fewer prostate cancer patients and those diagnosed longer ago did so compared to others, possibly because they were not asked to complete it, or they did not feel it necessary because they experienced little distress or less need for additional professional care [1,24,29,30]. Future research could examine patients’ and care providers’ barriers to instrument completion in oncology practice. Interestingly, patients who completed a DT&PL more often were more likely to recommend SDRN to others. This may suggest that patients increasingly understand the benefit of SDRN.

As for the provision of information, the vast majority of patients who completed a DT&PL indicated they received information about the purpose of SDRN and the DT&PL, and about referral options and the professional expertise of those they could be referred to (86% and 87% respectively). Adequate provision of information may increase patients understanding and motivation for adopting appropriate health interventions, such as SDRN. The relevance of providing information is reflected in the findings that opinions on SDRN and on the DT&PL were more positive in patients who received information on SDRN and the DT&PL and on availability and expertise of allied/psychosocial care providers.

Concerning patients’ experience with the fourth SDRN process step, namely communication, we found that DT&PL responses were discussed with three of four patients. This was lower than expected given that communication, regardless of the extent of distress, is an essential step in the Dutch SDRN process [22]. Possible explanations for this finding may be that care providers perceived a lack of time or skills [24,27,31]. Remarkably, 95% of the patients with whom responses were discussed were very satisfied and discussion of responses was most strongly related to recommending SDRN to others. These findings may encourage nurses to discuss responses.

Adequate referral is the fifth essential step in the SDRN process. This study suggests that once SDRN is implemented, it is patient need that dictates referral. Promisingly, only three of those indicating a need for care were not offered a referral; a major aim of distress screening is to ensure that cancer patients needing care are referred. Unfortunately, 25% of the respondents did not indicate whether referral was offered according to need. Of those who did not answer that question, 90% responded that the DT&PL was not discussed with them, while with 97% of those who did answer, responses were discussed. Thus, discussing responses seems instrumental in correctly identifying patients in need of additional care. This was also mentioned by the healthcare providers [24].

One-fifth of patients indicated a need for additional care at the time responses were discussed. This is comparable to some studies but lower than reported in other studies [32–35]. This range could be explained by the wording of the question. Studies with higher percentages ask about need in general terms [34,35], while those with lower percentages inquire more specifically about time or type of care [32,33].

All in all, patients’ evaluation of the DT&PL as a tool in the SDRN process is largely positive, though some patients were somewhat critical. This is comparable with studies investigating other instruments [17–19], and in line with care providers’ opinion on the DT&PL [24]. Notably, opinions on the DT&PL were also more positive in patients with whom responses were discussed, who were more satisfied with the discussion, and who would recommend SDRN to others. This suggests that incorporating the DT&PL in the SDRN process in a meaningful and clear manner, so that patients feel that instrument completion matters, has a positive effect.

In total, 78% of the respondents who completed a DT&PL would advise others to participate in SDRN. This percentage is somewhat lower than percentages found in (randomized) controlled intervention studies [14–16], but in RCTs much attention is given to ensure that the process is executed according to protocol for the duration of the trial. Our study reflects the less controllable situation in real world practice.

Overall, 61% of patients who completed a DT&PL encountered all steps in the process as recommended. We also found that patients are more positive about SDRN the more frequently they completed a DT&PL and the more all steps are followed. In fact, the likelihood of recommending SDRN to other patients increases with fuller exposure to the process. This finding underlines that care providers should adhere to all SDRN process steps, particularly to discussing DT&PL responses with all patients.

This study has a few limitations. The response rate was 54%, which is comparable to mailed questionnaire studies [36]. Due to study design, we cannot compare patients who participated to those who did not. Consequently, we cannot be certain that responders are not a selection of the total population approached. Representativeness and generalizability may be affected. Conclusions are based on the responses of the participating patients. Some of the hospitals implemented SDRN only for breast cancer patients; this is why breast cancer patients are overrepresented in the study. However, the sample is broad, including patients from various departments in 10 different hospitals, with wide variation on demographic and illness-related variables. The cross-sectional design, without control group, means that we cannot state conclusively that the SDRN process is the reason only 3 patients were not referred despite need. Regretfully, 20% of participants did not complete a DT&PL and thus could not answer all questions. This underlines the need for continuing attention to fully implementing SDRN. Also, it is unfortunate that patients who did not complete the DT&PL or with whom responses were not discussed were not asked about the reasons for this not happening.

## Conclusions

This study shows that, according to participating patients’ experiences a year after implementation, the majority encountered the steps of the SDRN process considered essential, with 3/5 having encountered all steps. Patients’ views on the DT&PL as tool are mainly positive. Patients’ perceived benefit of SDRN increases with fuller exposure to all steps, especially the discussion. After implementation, referrals are largely targeted to participating patients’ needs. Improvements in the execution of the SDRN process can be made, particularly in ensuring that all patients are regularly offered to complete a DT&PL and that responses are discussed with all patients.

## Supporting information

S1 File Questionnaire original and in english. (DOCX)

S2 File Dataset cancer patients experiences PLOSONE.7z Dataset cancer patients experiencesPLOSONE.7z Questionnaire original Questionnaire original. (7Z)
