# Supplementary material for: Cancer patients’ experiences with and opinions on the process 'Screening of Distress and Referral Need' (SDRN) in clinical practice: A quantitative observational clinical study
Source: PLoS One. 2018 Jun 14;13(6):e0198722. doi: 10.1371/journal.pone.0198722 (PMC6002053; doi:10.1371/journal.pone.0198722)
Supplement: S1 File — (DOCX) [file pone.0198722.s001.docx]

**S1 File. Questionnaire original and in english**

**Originele vragenlijst**

Deel 1a: Algemene gegevens

Wilt u de vragen invullen of het rondje invullen voor het antwoord wat voor u van toepassing is?

1. Wat is uw geboortedatum? - -
2. Datum waarop u deze vragenlijst invult - -
3. Wat is uw geslacht? Man

Vrouw

1. Wat is uw hoogst voltooide opleiding? Lager algemeen onderwijs, basisonderwijs

Lager beroepsonderwijs

Algemeen middelbaar beroepsonderwijs

Middelbaar beroepsonderwijs

Algemeen voortgezet onderwijs

Hoger beroepsonderwijs

Universitair onderwijs

**Deel 1b: vragen over uw ziekte**

1. Waar hebt u kanker gekregen?
   - Borst
   - Darm/rectum
   - Long
   - Nier/blaas
   - Penis
   - Baarmoeder(hals)/ eierstokken
   - Hoofd/hals
   - Slookdarm/maag
   - Schildklier
   - Lever
   - Weke delen/skelet
   - Huid
   - Hersenen
   - Prostaat
   - Lymfeklier
   - Anders, namelijk…………………….
2. Wanneer hebt u de diagnose gehoord? - -
3. Welke behandeling(en) hebt u (gehad)? Meerdere antwoorden mogelijk.
   - Operatie, op - -
   - Bestraling

Ben ik nog mee bezig Afgerond op - -

- - Chemotherapie

Ben ik nog mee bezig Afgerond op - -

- - Hormoontherapie

Ben ik nog mee bezig Afgerond op - -

- - Immunotherapie

Ben ik nog mee bezig Afgerond op - -

- - Actief afwachtend beleid (optie bij prostaatkanker)

**Deel 1c: vragen over de Lastmeter**

U hebt een of misschien meerdere keren de Lastmeter ingevuld. De Lastmeter is voor u een middel om hulpverleners te laten weten hoe het met u gaat en van welke klachten u last hebt en voor hulpverleners een hulpmiddel bij het gesprek met u. Graag horen wij uw mening over deze vragenlijst.

1. Hebt u na uw diagnose de Lastmeter (thermometer en lijst met mogelijke klachten) ingevuld?
   - Ja
   - Nee (einde vragenlijst)
2. Ik heb de Lastmeter tot nu toe ……… keer ingevuld.
3. Ik heb informatie ontvangen over het doel van psychosociale signalering en van de Lastmeter.
   - Ja
   - Nee
4. Hieronder staan uitspraken over invullen van de Lastmeter. Kruis aan wat voor u van toepassing is.

Invullen van de Lastmeter:

|  | Helemaal mee eens | Enigszins mee eens | Enigszins mee oneens | Helemaal mee oneens |
| --- | --- | --- | --- | --- |
| Is prettig | 1 | 2 | 3 | 4 |
| Is gemakkelijk | 1 | 2 | 3 | 4 |
| Is belastend | 1 | 2 | 3 | 4 |
| Is zinvol voor mijzelf | 1 | 2 | 3 | 4 |
| Is moeilijk | 1 | 2 | 3 | 4 |
| Is tijdrovend | 1 | 2 | 3 | 4 |
| Is geschikt voor het doel | 1 | 2 | 3 | 4 |
| Is zinvol voor mijn behandelaar | 1 | 2 | 3 | 4 |
| Geeft mij inzicht in de problemen die bij mij spelen | 1 | 2 | 3 | 4 |
| Helpt bij het bespreken van mijn problemen met mijn hulpverleners | 1 | 2 | 3 | 4 |
| Geeft mij inzicht in de ernst van mijn problemen | 1 | 2 | 3 | 4 |

1. a) Is de ingevulde Lastmeter met u besproken?
   - Ja
   - Nee (ga door naar vraag 9)

b) Met wie hebt u de resultaten van de Lastmeter besproken?

- - Met de specialist
  - Met de (oncologie)verpleegkundige
  - Met een andere zorgverlener

c) Hoe tevreden was u met dat gesprek?

- - Erg tevreden
  - Tevreden
  - Matig tevreden
  - Ontevreden

1. Is er naar aanleiding van uw antwoorden op de Lastmeter hulp aangeboden, die voldeed aan j uw behoefte?
   - Nee, er is mij geen hulp aangeboden en ik vond ook zelf dat ik geen hulp nodig had
   - Nee, ik had wel hulp gewild maar het werd me niet aangeboden
   - Ja, het werd me aangeboden maar ik had er zelf geen behoefte aan
   - Ja, ik wilde hulp en kreeg die ook
2. Ik heb informatie ontvangen over verwijsmogelijkheden en over de expertise van verschillende zorgprofessionals.
   - Ja
   - Nee
3. Zou u een ander met uw aandoening aanraden om de Lastmeter regelmatig in te vullen en met de behandelaar te bespreken
   - Ja
   - Nee

**Questionnaire in English**

**Part 1a: General**

1. Date of birth? - -
2. Date of questionnaire completion - -
3. Gender Male

Female

1. Highest level of education completed Elementary school

Low vocational education

High school

Secondary vocational education

High vocational education

University

**Part 1b: illness-related questions**

1. What type of cancer do/did you have?
   - Breast
   - Intestinal/rectal
   - Lung
   - Kidney/urologic
   - Penis
   - Gynaecologic
   - Head/neck
   - Esophagus/stomach
   - Thyroid
   - Liver
   - Sarcoma/bone
   - Skin
   - Brain
   - Prostate
   - Hematologic
   - Other, specifically …………………….
2. When were you diagnosed? - -
3. Which treatment(s) do/did you receive(d)? Multiple answers possible
   - Surgery, on - -
   - Radiotherapy

Ο Still ongoing Ο Finished on - -

- - Chemotherapy

Ο Still ongoing Ο Finished on - -

- - Hormone therapy

Ο Still ongoing Ο Finished on - -

- - Immunotherapy

Ο Still ongoing Ο Finished on - -

- - Watchful waiting (possible option for prostate cancer)

**Part 1c: questions on the Distress Thermometer and the Problem List (DT&PL)**

You have completed one or more Distress Thermometer and Problem List forms. This is a way for you to inform your healthcare providers about how you are doing and possible symptoms you are experiencing so that they can discuss this with you. We would like to know your opinion of this tool.

1. Have you completed the DT&PL since your diagnosis?
   - Yes
   - No (If no, this is the end of the questionnaire)
2. I have completed the DT&PL ..... times.
3. I have received information about the purpose of SDRN and the DT&PL
   - Yes
   - No
4. Below you see 11 statements about the DT&PL. Please circle the most appropriate answer.

DT& PL completion:

|  | Agree strongly | Agree | Disagree | Disagree strongly |
| --- | --- | --- | --- | --- |
| Is pleasant | 1 | 2 | 3 | 4 |
| Is easy | 1 | 2 | 3 | 4 |
| Is burdensome | 1 | 2 | 3 | 4 |
| Is useful for myself | 1 | 2 | 3 | 4 |
| Is difficult | 1 | 2 | 3 | 4 |
| Is time-consuming | 1 | 2 | 3 | 4 |
| Is suitable for its purpose | 1 | 2 | 3 | 4 |
| Is useful for my care provider | 1 | 2 | 3 | 4 |
| Offers me insight into problems I experience | 1 | 2 | 3 | 4 |
| Helps me in communication with my care provider | 1 | 2 | 3 | 4 |
| Gives me insight into the severity of my problems | 1 | 2 | 3 | 4 |

1. a) Was your completed DT&PL discussed with you?
   - Yes
   - No (go to question 9)

b) Who discussed your answers on the DT&PL with you?

- - Medical specialist
  - (Oncology) nurse
  - Another healthcare provider

c) How satisfied are you with that conversation?

- - Very satisfied
  - Satisfied
  - Moderately satisfied
  - Dissatisfied

1. Were you offered additional care based on your answers on the DT&PL?
   - No care was offered and I did not want additional care
   - No care was offered though I would have wanted additional care
   - Yes, care was offered, but I did not want it
   - Yes, care was offered and I wanted it
2. I have received information about referral options and the expertise of different healthcare providers.
   - Yes
   - No
3. Would you recommend that someone else with your illness complete the DT&PL and discuss their answers with a healthcare provider?
   - Yes
   - No
